# Effect of veneering material type and thickness ratio on flexural strength of bi-layered PEEK restorations before and after thermal cycling

**DOI:** 10.1007/s00784-022-04829-8

**Published:** 2023-01-05

**Authors:** Ahmed Gouda, Ashraf Sherif, Mennatallah Wahba, Tarek Morsi

**Affiliations:** 1 Postgraduate Student, Faculty of Oral and Dental Medicine, Future University in Egypt, Cairo, Egypt; 2 Fixed Prosthodontics Department, Faculty of Oral and Dental Medicine, Future University in Egypt, Cairo, Egypt; 3 Fixed Prosthodontics Department, Faculty of Dentistry, Ain Shams University, Cairo, Egypt; 4 Vice Dean Postgraduate Affairs. Faculty of Oral and Dental Medicine, Misr International University, Cairo, Egypt

**Keywords:** Flexural strength, PEEK, Thermocycling, Veneering

## Abstract

**Objective:**

The purpose of this study was evaluating the biaxial strength of bi-layered PEEK restorations before and after aging using different veneering materials in different thickness ratios.

**Material and Methods:**

Ninety specimens of thickness 1.5 mm were divided into three groups according to their veneering material. Group (CAD LD): BioHPP discs veneered with CAD milled lithium disilicate (*n*=30), group (CAD C): BioHPP discs veneered with CAD milled composite (*n*=30), and group (LC): BioHPP discs veneered with conventionally layered composite (*n*=30). Each group was subdivided into 3 subgroups (*n*=10) according to the different thickness ratios between the core and the veneering material (T_C:_T_V_). Subgroup 1: T_C:_T_V_=1:0.5, subgroup 2: T_C:_T_V_=0.7:0.8, and subgroup 3: T_C:_T_V_=0.5:1. Half of the specimens of each subgroup were subjected to thermocycling, and the bi-axial flexural strength of all specimens was tested before and after aging. Three-way ANOVA followed by Bonferroni’s post hoc test were used for data analysis. The significance level was set at *P* ≤ 0.05.

**Results:**

Material, thickness ratio, and aging all had a significant effect on biaxial flexural strength. (LC) group had the highest biaxial flexural strength. T_C:_T_V_=0.5:1 showed the lowest biaxial flexural strength. All groups showed significant decrease in biaxial flexural strength after aging.

**Conclusions:**

Veneering material for PEEK together with the thickness ratio between the core and veneering material greatly affect the flexural strength of bi-layered restorations. Thermocycling negatively impacts the flexural strength of PEEK bi-layered restorations.

**Clinical significance:**

According to the results of that study, PEEK cores are best veneered with conventionally layered composite with core to veneering thickness ratio being 1:0.5.

## Introduction

Polyetheretherketone (PEEK) is a member of the polyaryletherketone polymer family. It is a polycyclic, aromatic, thermoplastic polymer that is semi-crystalline and has a linear structure. PEEK has superior mechanical properties to zirconia and lithium disilicate. Fracture resistance of PEEK three-unit fixed dental prosthesis is reported to be 1200–1383 N which is higher than that of zirconia and lithium disilicate glass-ceramic with that of the former is 981–1331 N and that of the later is 950 N. Additionally, PEEK is light in weight, has high resistance to chemical wear, and is a biologically inert material that is highly compatible with the surrounding tissue [1, 2].

One of the most significant properties of PEEK is its low elastic modulus of 3.6 GPa. Through the addition of reinforcing agents such as carbon fibers, such elastic modulus can be improved to 18 GPa which is considered close to that of cortical bone and dentin. On the other hand, zirconia displays modulus of elasticity of 220 GPa, while that of lithium disilicate is 95 GPa [3, 4]. A 3D-finite element analysis of monolithic full contour posterior crowns revealed that materials with higher elastic modulus present higher tensile stress concentration on the crown intaglio surface and higher shear stress on the cement layer that could facilitate crown debonding in oral conditions [5].

A modified PEEK material containing 20% ceramic filler has been introduced to the dental market as BioHPP. BioHPP is a PEEK variant exhibiting excellent chemical, mechanical, and thermal properties. It has been especially optimized for dental use. It contains ceramic micro-particles such as aluminum oxide and zirconium oxide for optimization of the mechanical properties, better degree of polishability, reduction of plaque retention, and higher color stability over time [6, 7].

Regardless of its good mechanical properties, the use of BioHPP as a monolithic restoration is limited owing to its high opacity, greyish to white color, and low translucency compared to dental ceramics. Consequently and for the time being, its use with additional veneering is crucial [8].

In literature, core veneered restorations have been deemed as the cornerstone for prosthetic dentistry where the combination of a strong core together with an esthetic veneer has proven successful for many decades. While veneering materials aim to rebuild the outer layer of the restoration, a core material is required to strengthen the integrity and stability of the restoration [9, 10]. Consequently, BioHPP, with its high strength, and modulus of elasticity comparable to that of bone and dentin, yet less than satisfactory optical properties, has the potential to be used as a core material for esthetic restorations while being veneered by a variety of materials and techniques. Multiple studies have investigated the effect of such different combinations between PEEK and veneering materials on the mechanical performance of those bi-layered restorations, yet data is still limited [11–14].

However, mechanical performance of a bi-layered restoration depends not only on the nature of the veneering material used, but also on the thickness of the restoration and the thickness ratio between the core and the veneering material. Most of previous studies that evaluated the effect of core thickness and failure mode of bi-layered dental restorations showed that variations in the core thickness could significantly influence the flexural strength as well as the failure mode of the bi-layered restorations with a thicker core generally yielding a higher flexural strength and a thicker veneer yielding a lower value [15, 16].

A fact not to be overseen while evaluating the mechanical performance of the restoration is that such dental restorations are exposed in the oral environment to different destructive stimuli such as mastication, the effect of water, and temperature fluctuations. Currently, there is limited data concerning the effect of aging on strength properties of bi-layered PEEK restorations [12, 14, 17, 18].

In vitro simulations can be useful to predict the longevity of dental materials, evaluating their mechanical and physical behavior during clinical aging. In the field of laboratory research, thermal cycling is one of the most widely used and highly accepted procedures to simulate the in vivo aging of restorative materials. It is based on subjecting the tested materials to repeated cyclic exposures of hot and cold temperatures, in a water baths attempting to reproduce the thermal changes found in the oral cavity [18].

To date, only limited information is available on the effect of aging together with using different veneering materials of PEEK as well as the effect of varying the thickness ratio between the PEEK core and the veneering materials on the flexural strength of PEEK veneered restorations. Consequently, our goal in this study was to evaluate the effect of different veneering materials of PEEK as well as the effect of varying the thickness ratio between the PEEK core and the veneering materials on the flexural strength of bi-layered restorations before and after aging. The null hypothesis was that neither of those three factors would have an effect on flexural strength.

## Materials and methods

A total of ninety disc-shaped bi-layered PEEK specimens of diameter 12 mm and thickness 1.5 mm were constructed and randomly divided into three groups (*n*=30) according to the type of the veneering material used. Each group was further subdivided into 3 subgroups according to the thickness ratio (T_C:_T_V_) between the PEEK core and the veneering material (*n*=10) (Tables 1 and 2). Although the manufacturer’s recommendations for BreCAM BioHPP minimum thickness when used as a framework material is 0.7 mm [19–21], the authors chose to study the effect of different framework thicknesses in addition to that recommended by the manufacturer while keeping the total thickness fixed to 1.5 mm as recommended for most of the esthetic bi-layered restorations [15, 22].

**Table 1:** Sample grouping

Veneering material	Core-to-veneer thickness ratio (T_C_:T_V_)						
	T_C_:T_V_ =1:0.5 (*n*=10)		T_C_:T_V_ =0.7:0.8 (*n*=10)		T_C_:T_V_ =0.5:1 (*n*=10)		Total
IPS.e max (CAD LD)	Before aging	After aging	Before aging	After aging	Before aging	After aging	*n* =30
	*n* =5	*n*=5	*n* =5	*n* =5	*n* =5	*n* =5	
HIPC (CAD C)	Before aging	After aging	Before aging	After aging	Before aging	After aging	*n* =30
	*n* =5	*n* =5	*n* =5	*n* =5	*n* =5	*n* =5	
Crea Lign (LC)	Before aging	After aging	Before aging	After aging	Before aging	After aging	*n* =30
	*n* =5	*n* =5	*n* =5	*n* =5	*n* =5	n =5	
							*n* =90

**Table 2:** Materials used in the study

Group	Material	Type	Composition	Manufacturer
1	Bre.CAM BioHPP	Modified polyetheretherketone	• Polyetheretherketone 80% • Aluminum oxide & zirconium Oxide 20%	bredent GmbH & Co KG
2	Bre.CAM HIPC	High impact polymer composite	• Amorphous cross-linked polymethyl methacrylate matrix Micro-ceramic fillers	bredent GmbH & Co KG
3	Crea.lign	Light cured composite	• High strength Oligomer matrix • Nano-ceramic fillers 50%	bredent GmbH & Co KG
7	IPS e.max CAD	Lithium disilicate (CAD-milled)	SiO_2_ 57.0–80.0 %, Li_2_O 11.0–19.0%, K_2_O 0.0–13.0%, P_2_O_5_ 0.0–11.0%, ZrO_2_ 0.0–8.0%, ZnO 0.0–8.0%, other and coloring oxides 0.0–12.0%	Ivoclar Vivadent

First, Bre.CAM.BioHPP (bre.CAM.BioHPP; bredent GmbH & Co KG), IPS e.max CAD (Ivoclar Vivadent, Schaan, Liechtenstein), and Bre.CAM HIPC (bre.CAM.BioHPP; bredent GmbH & Co KG) discs were cut and prepared. Ninety discs of Bre.CAM.BioHPP of diameter 12 mm and thicknesses 0.5 mm, 0.7 mm, and 1 mm, as well as thirty discs of Bre.CAM HIPC of diameter 12 mm and thicknesses 0.5 mm, 0.8 mm, and 1 mm, were cut from their respective blanks. Disc design was made on Exocad Dental CAD software (Exocad GmbH, Germany), saved as STL file, and was then exported for milling using SHERA ecomill 5-axis milling machine (SHERA Technology GmbH, Germany).

Discs of both materials were finished wet using waterproof silicon carbide sandpaper (Matador SoftFlex, Germany) of different grit sizes ranging from 320 to 1200, and the final thickness was checked with electronic digital caliper (IP54, Jiangsu, China). All discs were sandblasted on one side according to manufacturer’s instructions by 110 microns aluminum oxide powder basic quattro (Renfert, Hilzingen, Germany) at an angle of 90° and distance 3 cm for 10 s using a cylindrical metal holding device for distance standardization. Discs were then ultra-sonically cleaned in an ultrasonic bath (L&R, Kearny, NY, USA) filled with deionized water for 5 minutes. Sandblasted surface of all discs was coated with a single layer of Visio.link (Bond.lign; bredent GmbH & Co KG) and light cured for 90 s at a wavelength of 370–500 nm using bre. Lux Power Unit (bredent GmbH & Co KG) for priming the surface of Bre.CAM. BioHPP.

For preparation of IPS e.max CAD veneered specimens, partially crystalized IPS e-max CAD blocks were first shaped into a cylindrical form of 12 mm diameter, where a cylinder design was made on Exocad Dental CAD and saved as STL file that was used for milling the IPS e.max CAD block using wet milling machine Arum version 5x-400 (Daejaeon, Republic of Korea). Thirty discs were then cut from their respective cylinder with diameter of 12 mm and 0.5, 0.8, and 1 mm thickness using IsoMet 4000 micro saw (Isomet 4000 precision cut, Buchler. USA) under water coolant, thickness was then verified using an electronic digital caliper, and then all discs were crystallized in EP3010 ceramic furnace (Ivoclar Vivadent, Schaan, Liechtenstein ) according to the manufacturer’s instructions. All IPS.emax CAD discs were acid etched according to the manufacturer’s instructions using Bisco hydrofluoric acid etch 9.5% (BISCO, USA) for 20 s, washed, air dried with oil-free air, and salinized with Bisco Porcelain Primer (BISCO, USA) for 60 s.

To obtain a bi-layered specimen for groups (CAD LD) and (CAD C), the Bre.CAM BioHPP disc was placed, with its treated surface facing upward in an especially designed round shaped cobalt chrome mold of dimensions 12 mm diameter × 1.56 mm thickness designed to accommodate the total specimen thickness of 1.5 mm leaving an additional 0.06 μm for cement thickness [9, 12].

A layer of Duo-Link (BISCO, USA) dual cured resin cement of universal shade was applied on the treated Bre.CAM BioHPP surface, and the IPS.emax CAD disc for (CAD LD) group or HIPC disc for (CAD C) group of the corresponding thickness was placed over it with its treated surface facing the cement surface.

To provide a uniform thickness of the luting cement, the mold—with the bi-layered specimen in it—was placed between two glass plates, and a loading device with a 5 kg weight was applied for 10 mins standardizing the pressure applied over the specimen [9]. After weight removal, further polymerization was carried out at a wavelength of 370–500 nm using bre. Lux Power Unit for 180 s, and the veneer surface of each specimen was polished according to manufacturer’s instructions using OptraFine ceramic polishing system (Ivoclar Vivadent, Schaan, Liechtenstein) for (CAD LD) specimens and Visio.lign Toolkit (Bredent GmbH, Germany) for (CAD C) specimens for 15 s with a low-speed handpiece under water coolant.

For (LC) specimens, each Bre.CAM BioHPP disc was placed in the especially designed mold with its treated surface facing upward, and one uniform layer of Crea.lign opaquer (Bredent GmbH, Germany) was layered on the Bre.CAM BioHPP discs and polymerized for 360 s with the bre. Lux Power Unit. A3 dentin shade Crea.lign paste (Bredent GmbH, Germany) was applied, and the mold was pressed between two glass plates to obtain a smooth, bubble-free surface of uniform thickness before polymerization. The polymerization was carried out for 360 s using bre. Lux Power Unit. Finally, each specimen was polished using the Visio.lign Toolkit for 15 s in a low-speed handpiece.

All specimens in all groups were washed with air/water spray for 10 s to remove any polishing residues, and the final thickness of the bi-layered discs was verified using an electronic digital caliper.

Half of the specimens of each subgroup were tested for biaxial flexural strength using piston-on-three-balls test as described in the ISO standard 6872 for dental polymers and ceramics. Specimens were placed concentrically on the supporting balls, and load was applied with a universal testing machine (Instron Co., Canton, Mass.), through a flat punch of tip diameter 2 mm at cross-head speed 1 mm/min with the core surface being placed in tension for all specimens. The load at the point of fracture was recorded, and biaxial flexural strength was calculated through the following equation given in the ISO 6872 [23, 24];

$$\sigma =\frac{6\textrm{M}}{{\mathrm{t}}_{\textrm{a}}^2{\textrm{K}}_{2p}}\left[\frac{{\textrm{E}}_{\textrm{b}}{\mathrm{t}}_{\textrm{b}}\left(1\hbox{-} {v}_{\textrm{a}}^2\right)}{{\textrm{E}}_{\textrm{b}}{\mathrm{t}}_{\textrm{b}}\left(1\hbox{-} {v}_{\textrm{a}}^2\right)}+\frac{{\textrm{t}}_{\textrm{a}}\left(1\hbox{-} {v}_{\textrm{a}}^2\right)\left(1+\frac{{\textrm{t}}_{\textrm{b}}}{{\textrm{t}}_{\textrm{a}}}\right)\left(1+\frac{{\textrm{E}}_{\textrm{a}}{\textrm{t}}_{\textrm{a}}}{{\textrm{E}}_{\textrm{b}}{\textrm{t}}_{\textrm{b}}}\right)}{{\textrm{t}}_{\textrm{b}}{\left(1+\frac{{\textrm{E}}_{\textrm{a}}{\textrm{t}}_{\textrm{a}}}{{\mathrm{E}}_{\textrm{b}}{\mathrm{t}}_{\textrm{b}}}\right)}^2\hbox{-} {\left({v}_{\textrm{a}}\frac{v_{\textrm{b}}{\mathrm{E}}_{\textrm{a}}{\mathrm{t}}_{\textrm{a}}}{{\mathrm{E}}_{\textrm{b}}{\mathrm{t}}_{\textrm{b}}}\right)}^2}\right]$$

$${\textrm{K}}_{2p}=1+\frac{{\textrm{E}}_{\textrm{b}}{\textrm{t}}_{\textrm{b}}^3\left(1-{v}_a^2\right)}{{\textrm{E}}_{\textrm{a}}{\mathrm{t}}_{\textrm{a}}^3\left(1-{v}_{\textrm{b}}^2\right)}+\frac{3\left(1-{v}_{\textrm{a}}^2\right){\left(1+\frac{{\textrm{t}}_{\mathrm{b}}}{{\textrm{t}}_{\mathrm{a}}}\right)}^2\left(1+\frac{{\textrm{E}}_{\mathrm{a}}{\textrm{t}}_{\mathrm{a}}}{{\textrm{E}}_{\mathrm{b}}{\textrm{t}}_{\mathrm{b}}}\right)}{{\left(1+\frac{{\textrm{E}}_{\mathrm{a}}{\textrm{t}}_{\textrm{a}}}{{\mathrm{E}}_{\textrm{b}}{\mathrm{t}}_{\textrm{b}}}\right)}^2-{\left({v}_{\textrm{a}}+\frac{v{\textrm{b}\mathrm{E}}_{\textrm{a}}{\mathrm{t}}_{\textrm{a}}}{{\mathrm{E}}_{\textrm{b}}{\mathrm{t}}_{\textrm{b}}}\right)}^2}$$

where (*σ*) is the bi-axial flexural stress; (*t*_a_) and (*t*_b_) are thicknesses of the two material layers where (a) is the material on top and (b) is the material at the bottom; *E*_a_ and *E*_b_ are the are the Young’s modulus of the core and veneer layers, respectively. (*υ*) is Poisson’s ratio and is taken as the mean value between the Poisson’s ratios of veneering material and BioHPP core. (M) is the maximum bending moment calculated from the equation:

$$\textrm{M}=\frac{\textrm{W}}{4\pi}\left[\left(1+v\right)\log \frac{\textrm{A}}{\mathrm{R}}+1\right]$$

where (*W*) is the load, (*R*) is the equivalent radius of loading, and (*A*) is the radius of the circle of the support points which is 5 mm and

$$\mathrm R=\;\surd1.6\mathrm B^2+\mathrm d^2\;-0.675\mathrm d$$

where (*B*) is the radius of the tip of the piston which is 1 mm and (*d*) is the thickness of the specimens which is 1.56.

The other half of the specimens in each subgroup was subjected to artificial aging through thermal cycling furnace (julabo. THE-1100) at temperature range between 5 and 55 °C and a dwell time of 30 s for 5000 cycles simulating 6 months of intra-oral service [8, 12, 18, 25, 26]. After thermal cycling, biaxial flexural strength was tested in the same way.

Numerical data was explored for normality by checking the distribution of data and using tests of normality (Kolmogorov-Smirnov and Shapiro-Wilk tests). Biaxial flexural strength data showed parametric (normal) distribution. Data were presented as mean and standard deviation (SD) values. Three-way ANOVA test was used to study the effect of material, thickness ratio, and aging as well as their interactions on biaxial flexural strength. Bonferroni’s post hoc test was used for pair-wise comparisons when ANOVA test is significant. The significance level was set at *P* ≤ 0.05. Statistical analysis was performed with IBM SPSS Statistics for Windows, Version 23.0. Armonk, NY:IBM Corp.

Finally, all specimens surfaces were examined under a stereoscope ×20 (Leica MZ 6 stereomicroscope; Leica Microsystems, Germany), and then a representative specimen from each group was observed with a ZEISS field emission scanning electron microscope (SEM) (Carl Zeiss Microscopy GmbH Germany) after sputter-coating with gold at 2 kV with a working distance of 5.0–6.0 mm.

## Results

Kolmogorov-Smirnov and Shapiro-Wilk tests of normality showed no evidence of violation of normality of data distribution. According to the three-way ANOVA, the results showed that the veneering material, the ratio between the core and veneer thickness, and aging as well as the interaction between them (*P* value <0.001) had a significant effect on the biaxial flexural strength of the tested specimens as shown in (Table 3).

**Table 3:** Three-way ANOVA results for the effect of different variables on mean biaxial flexural strength (MPa)

Source of variation	Type III sum of squares	df	Mean square	*F* value	*P* value	Effect size *(partial eta squared)*
Material	239253.1	2	119626.5	176.293	<0.001*	0.83
Thickness ratio	50862.6	2	25431.3	37.478	<0.001*	0.51
Aging	290936.1	1	290936.1	428.752	<0.001*	0.856
Material × thickness ratio × aging interaction	11908.7	4	2977.2	4.387	0.003*	0.196

df: degrees of freedom = (n-1); *significant at P ≤ 0.05

Bonferroni’s post hoc test for pair-wise comparisons revealed that (CAD C) group showed the highest mean biaxial flexural strength values (446.6 MPa) and (363.2 MPa) with the thickness ratios of T_C:_T_V_= 1.0.5 and T_C:_T_V_= 0.5:1, respectively. On the other hand, (CAD LD) group showed the lowest mean biaxial flexural strength values (197.9 MPa) and (235 MPa) with the thickness ratios of T_C:_T_V_= 1.0.5 and T_C:_T_V_= 0.7:0.8, respectively (Table 4).

**Table 4:** The mean, standard deviation (SD) values, and Bonferroni’s post hoc test results for pair-wise comparison between biaxial flexural strength (MPa) of the three materials with different thickness ratios before and after aging.

Aging	Thickness ratio T_C_:T_V_	IPS e.max CAD (CAD LD)		HIPC CAD (CAD C)		Crea.lign (LC)		*P* value
		Mean	SD	Mean	SD	Mean	SD	
Before aging	1:0.5 mm	197.9 ^C^	14	377.3 ^B^	17.9	446.8 ^A^	24.7	<0.001*
	0.7:0.8 mm	235 ^B^	39.9	388.3 ^A^	34.1	379.5 ^A^	36	<0.001*
	0.5:1 mm	296.1 ^B^	37.6	193 ^C^	32.3	363.2 ^A^	42	<0.001*
After aging	1:0.5 mm	88.3 ^C^	10.2	210.8 ^B^	6.4	300.7 ^A^	23.2	<0.001*
	0.7:0.8 mm	198.1 ^B^	6.6	246.7 ^A^	27.6	277.1 ^A^	13.4	<0.001*
	0.5:1 mm	208.4 ^A^	16.2	113.4 ^B^	23.2	209.9 ^A^	18.6	<0.001*

*Different superscripts in the same row indicate statistically significant difference between materials*

*Significant at P ≤ 0.05

Aging caused statistical significant decrease (*P* value <0.001) in biaxial flexural strength for all materials and all thickness ratios (Table 5).

**Table 5:** The mean, standard deviation (SD) values, and results of three-way ANOVA test for comparison between biaxial flexural strength (MPa) before and after aging with different materials and thickness ratios

Material	Thickness ratio	Before aging		After aging		*P* value	Effect size *(partial eta squared)*
		Mean	SD	Mean	SD		
IPS e.max CAD	1:0.5 mm	197.9	14	88.3	10.2	<0.001*	0.38
	0.7:0.8 mm	235	39.9	198.1	6.6	0.028*	0.065
	0.5:1 mm	296.1	37.6	208.4	16.2	<0.001*	0.282
HIPC CAD	1:0.5 mm	377.3	17.9	210.8	6.4	<0.001*	0.587
	0.7:0.8 mm	388.3	34.1	246.7	27.6	<0.001*	0.506
	0.5:1 mm	193	32.3	113.4	23.2	<0.001*	0.245
Crea.lign	1:0.5 mm	446.8	24.7	300.7	23.2	<0.001*	0.522
	0.7:0.8 mm	379.5	36	277.1	13.4	<0.001*	0.349
	0.5:1 mm	363.2	42	209.9	18.6	<0.001*	0.546

*Significant at P ≤ 0.05

**Fig. 1:**
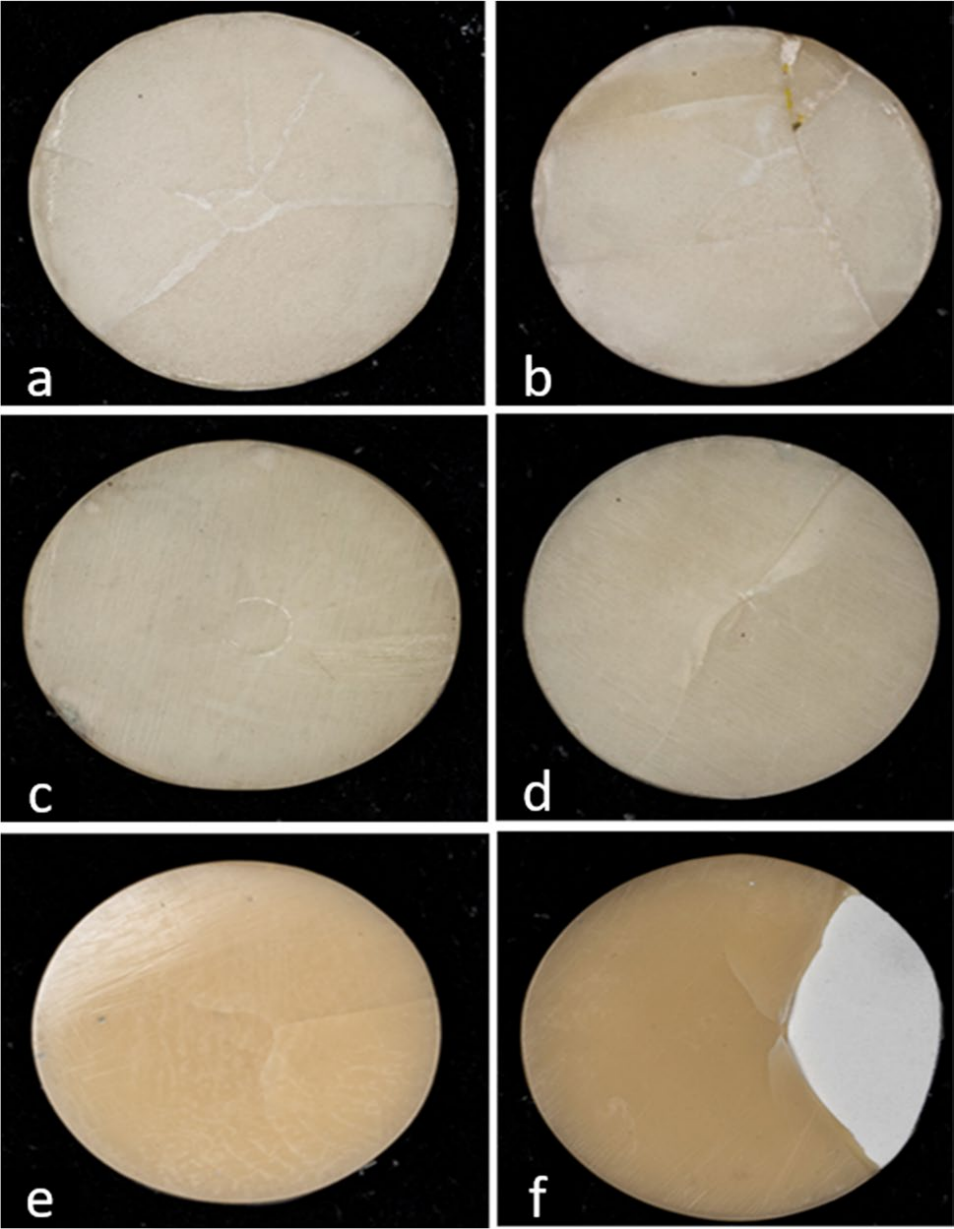
Digital microscopic assessment (×20) of T_C:_T_V_ = 1:0.5 mm demonstrating: **a** IPS emax specimen before aging; **b** IPS emax specimen after aging; **c** HIPC specimen before aging; **d** HIPC specimen after aging; **e** Crea.lign specimen before aging; **f** Crea.lign specimen after aging

**Fig. 2:**
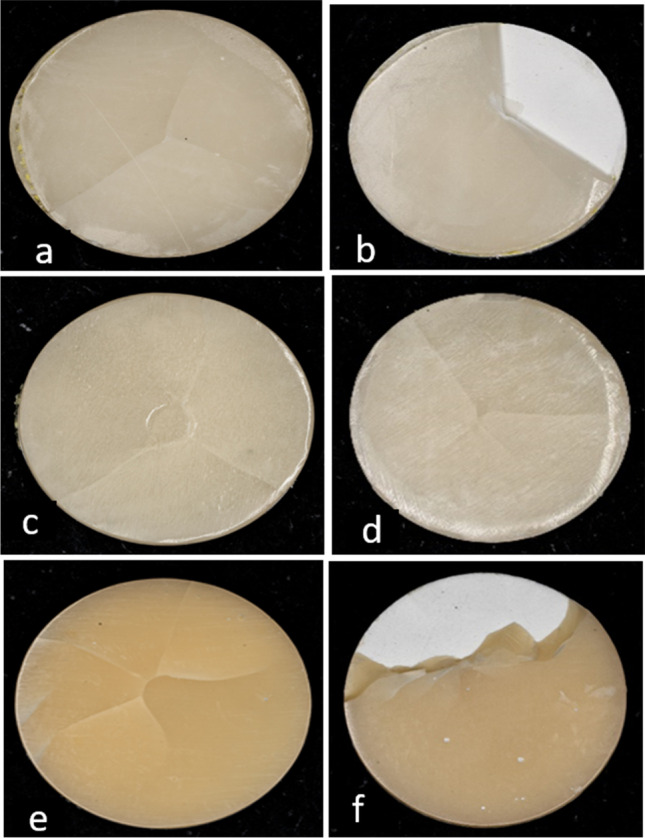
Digital microscopic assessment (×20) of T_C:_T_V_ = 0.7:0.8 mm demonstrating: **a** IPS emax specimen before aging, **b** IPS emax specimen after aging, **c** HIPC specimen before aging, **d** HIPC specimen after aging, **e** Crea.lign specimen before aging, **f** Crea.lign specimen after aging

**Fig. 3:**
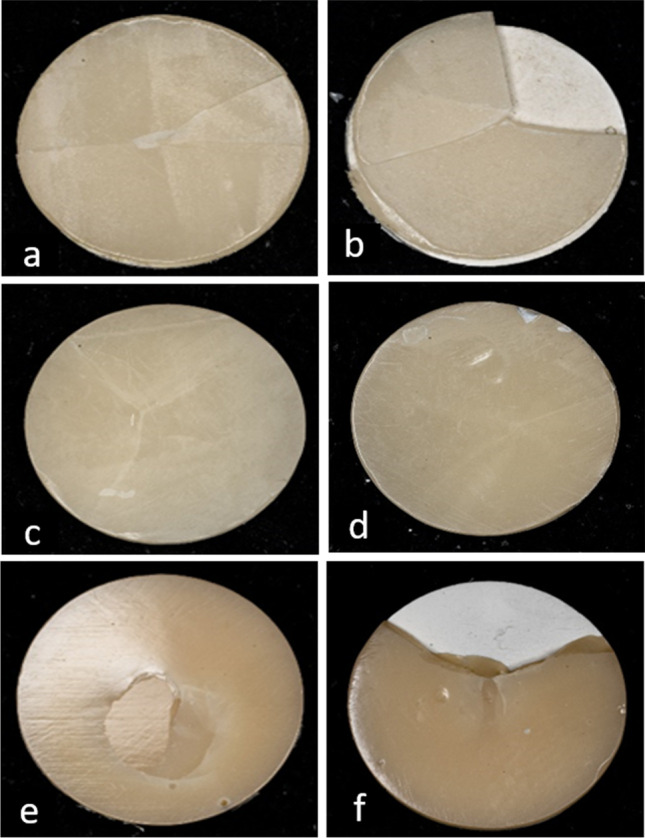
Digital microscopic assessment (×20) of T_C:_T_V_ = 0.5:1 mm demonstrating: **a** IPS emax specimen before aging, **b** IPS emax specimen after aging, **c** HIPC specimen before aging, **d** HIPC specimen after aging, **e** Crea.lign specimen before aging, **f** Crea.lign specimen after aging

The fracture type analysis (Figs. 1, 2, and 3) showed that the fracture type was comparable and showed cracks in the veneering material for all the un-aged specimens, but was less intense in the (CAD C) group. In the aged groups, there was evidence of completely exposed PEEK surface for all materials and thickness ratios.

**Fig. 4:**
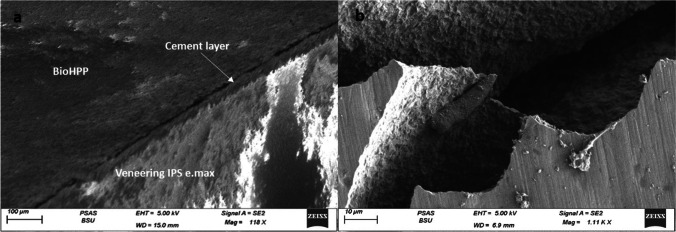
SEM fractograph for IPS emax CAD specimen: **a** low magnification showing debonding at the cement interface, **b** higher magnification demonstrating sharp fractures in the veneering material extending down to the BioHPP and exposing it

**Fig. 5:**
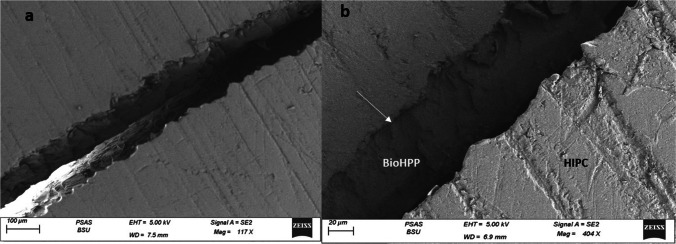
SEM fractograph for HIPC specimen demonstrated a fracture in the veneering material. **a** Low magnification showing a crack extending through the veneering HIPC itself and exposing the BioHPP core. **b** Higher magnification with the white arrow showing a debonding interface

**Fig. 6:**
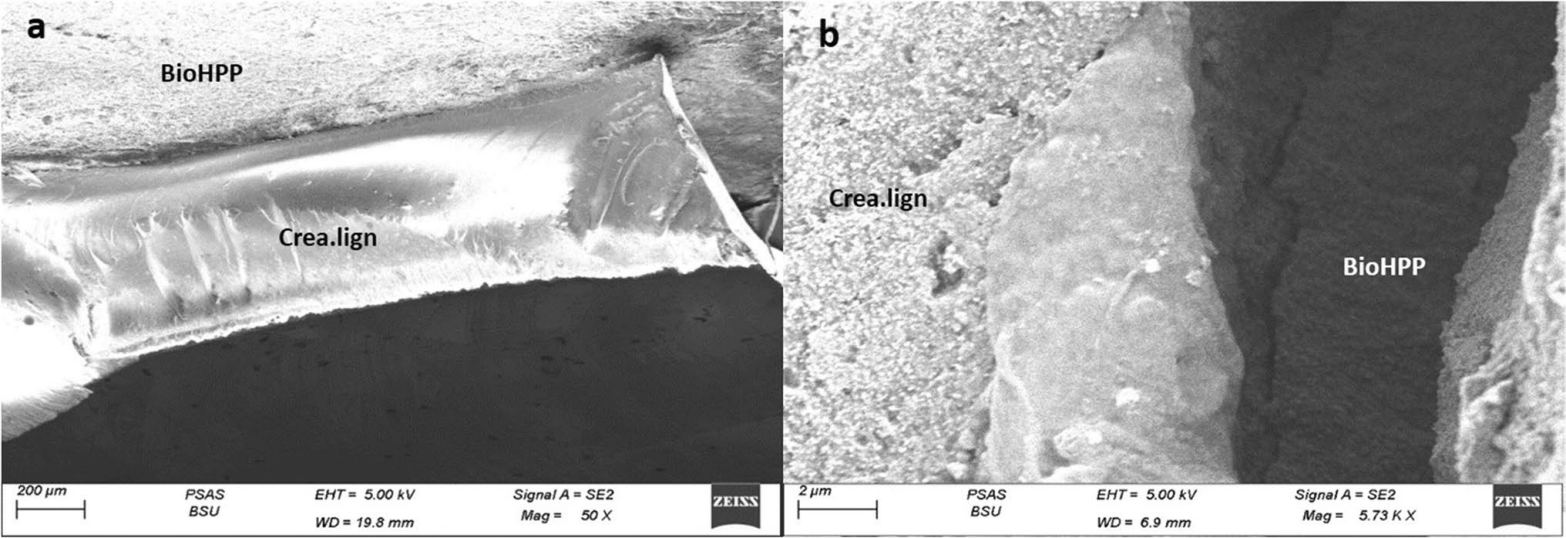
SEM fractograph for Crea.lign specimen: **a** low magnification showing the interface area between the veneering material and BioHPP revealing good bonding and limited areas of debonding, **b** higher magnification demonstrating fracture in the veneering material extending down to the BioHPP core

SEM analysis of the surfaces of the representative fractured specimens after the biaxial flexural strength test showed that most of specimens suffered from a mixed adhesive/cohesive failure. The representative specimen for the (CAD LD) group demonstrated sharp fractures in the veneering IPS e.max CAD extending down to the BioHPP core and exposing it with a clear demarcation line at the cement interface denoting debonding (Fig. 4). As for the (CAD C) group representative specimen, there was an evident crack extending through the veneering HIPC itself and exposing the the BioHPP core (Fig. 5). Finally, the specimen from (CAD LC) group displayed a fracture line extending in both the veneering Crea.lign and the BioHPP core with exposure of the underlying core (Fig. 6).

## Discussion

This in vitro study evaluated the effect of veneering material type, core/ veneer thickness ratio as well as the effect of aging on the biaxial flexural strength of bi-layered PEEK restorations. The null hypothesis was rejected as there was significant effect (*P* value <0.001) for all the three factors; veneering material, thickness ratio of the core and veneer, and aging on the biaxial flexural strength values.

In recently published data**,** Sloan et al. [11] reported that bonded BioHPP to lithium disilicate had low flexural strength and attributed this to the nature of bonding to PEEK substrate that is thought to be almost entirely micromechanical because of the unreactive nature of the PEEK surface. Additionally, an earlier research by Taufall et al. [14] stated that regardless of the fact that the veneering material might be having a high strength, the reason behind the adhesive failure is the pre-treatment. They claimed that although airborne-particle abrasion could improve the micro-roughness and increase the surface area allowing better infiltration of the adhesive material, the bonding still remains almost entirely mechanical between the PEEK surface and the adhesive. However, it was suggested that that pentaerythritol triacrylate (PETIA) in Visio.link has a high ability to modify the PEEK surface, thus providing higher bonding properties where the veneering material is chemically bonded to the adhesive Visio.link [13]. On the other hand, it has been previously documented that light cured adhesives and dual cured resin cements, when used together, could have a significantly low bond strength. This reduced bond strength arises from the incompatibility between the acidic resin monomer employed in the adhesive and the peroxide amine catalyst incorporated in the dual cured resin [27]. Our results are seen to resonate with the previously mentioned work where results revealed that groups manually layered with conventional composite resin showed the highest mean biaxial flexural strength values while CAD-milled lithium disilicate veneered groups showed the lowest mean biaxial flexural strength. Based on the aforementioned data, it could be assumed that bond strength was less than ideal in (CAD LD) and (CAD C) groups where the bond was created between the light cured Visio.link and the dual-cured resin cement. On the other hand, in group (LC), a stable chemical bond was generated between the Visio.link adhesive and light cured composite resin. Such assumption could also be supported through our SEM analysis where representative specimens from both (CAD LD) and (CAD C) showed a demarcation line denoting areas of debonding at the cement interface.

Furthermore, the higher biaxial flexural strength values of BioHPP when veneered with the composite resin, whether CAD-milled or conventionally layered could be rendered to the intrinsic mechanical behavior of BioHPP as well as the veneering materials where it has been documented in literature that materials that have more compatible elastic modulus tend to bend under load and distribute stresses more evenly. Thus, BioHPP with its modulus of elasticity being close to both CAD-milled HIPC composite and conventionally layered Crea.lign might be a reason to reduce stress induction at the different layers interfaces of the specimens [28]. On the other hand, when BioHPP was veneered with a rigid material having a different modulus of elasticity such as CAD-milled lithium disilicate, stress concentration occurred at the critical area of the cement causing failure [29, 30].

When comparing the two composite veneered groups, groups manually veneered with conventional composite displayed the highest flexural strength values which come in accordance with the results of Beleidy et al. [12]. Such higher flexural strength values could be due to the influence of the modulus of elasticity of the examined veneering materials. PEEK as a framework has a modulus of elasticity of 4.0 GPa while that of HIPC and Crea.lign is 2.8 GPa and 4.4 GPa, respectively [14]. Moreover, Crea.lign composite has a 50 % nano-ceramic fillers content where it has been documented that adding nano-filler particles to the resin matrix of dental composites improves its mechanical properties [30].

However, it was clear in our study that different veneering materials behaved differently when considering different thickness ratios between the BioHPP core and the veneering material though all of them showed comparable cracking failure type when observed under the digital stereomicroscope.

Previously obtained data of clinically failed all-ceramic restorations suggested that the fracture origin is located at the tensile surface of the restoration that is the BioHPP core in our study. It was stated that strength, reliability, and mode of fracture of bi-layered structures are determined by the material on the bottom surface under biaxial tensile stress [31, 32]. Such findings from literature explain well the reason why increasing the thickness of the BioHPP core showed higher mean biaxial flexural strength for both the conventionally layered and CAD milled composite veneered groups.

In contrast, IPS e.max CAD veneered groups showed significant increase in flexural strength values as the veneering material thickness increased: a finding that could be multi-factorial. The effect of the bond quality between BioHPP substrate and lithium disilicate being almost entirely micromechanical, the inherent flexural strength of lithium disilicate being 360 MPa, the difference in modulus of elasticity between BioHPP and the veneering lithium disilicate, and the fracture toughness and its correlation with thickness are all assumed to play a role [14, 28, 33].

Millen et al. [34] showed in a previous study that bi-layered restorations with thinner copings and thicker veneering material display an increase in fracture toughness, which is a quantitative measure of the ability of a material to resist propagation of a pre-existing crack. This in turn contributes to lowering the residual stress within the bi-layered system. In another study, Graupner N et al. [35] stated that a highly brittle material shows higher toughness with larger sample thickness, a behavior that seems to correlate with the elongation at break.

Additionally, one more possible reason explaining the behavior of our lithium disilicate veneered group specimens is the past data obtained from fractographic analysis of clinically failed specimens showing that failure is thought to begin when compressive forces on the load contacting surface result in bending at the cement interface. This tensile stress arising from differences in the elastic moduli of ceramic and substrate results in bending at the cement interface and eventually leads to crack initiation [33, 36].

Regardless of the effect of the veneering material or the thickness ratio of the core and the veneer, when all groups were subjected to thermal cycling in an attempt to simulate the oral conditions, the results showed that thermal cycling had a significant negative impact on biaxial flexural strength. Those results, however, contradict what was claimed by Xin et al. [18] and Taufall et al. [14] that thermal cycling had no effect on flexural strength or load bearing capacity of PEEK.

Although literature has been contradicting about the effect of thermal cycling [13], our results are consistent with results obtained by Beleidy et al. [12] who showed a significant lower fracture resistance for both HIPC veneered PEEK cores and manually layered Crea.lign composite veneered PEEK cores after aging.

That negative impact of thermal cycling was evident also through the digital microscopic analysis where images showed clear exposure of some parts of the BioHPP surface in all groups after thermal cycling suggesting an adhesive failure [14]. Such findings could be reverted to the negative effects thermal cycling have on the composite resin material that is presented in our study as either the resin cement used with both (CAD LD) and (CAD C) groups or the veneering Crea.lign composite resin in (LC) group. In literature, multiple studies have reported molecular interactions occurring between water and the epoxy network when a resinous material is subjected to thermal cycling. When some positive ions in the filler particles react with water, a hydrolytic degradation takes place which thus changes the charge balance of the silica matrix. When the number of hydrogen ions occupying the free spaces increase, the (Si-O-Si) bonds of the silica matrix break down, and surface degradation occurs [33]. Moreover, thermal cycling was also reported to cause water sorption into the epoxy network and swelling of the matrix with water taken up by the polymer network causing filler-matrix debonding. Such debonding can affect the water penetration mechanisms creating new channels for the moisture access resulting in resin softening and hydrolytic degradation of fillers. Consequently, it is suggested that water acts like a plasticizer that could weaken the polymer network directly by causing the breakdown of chemical bonds of the silane-filler interface and filler particle surfaces [37, 38].

These interactions negatively influence the thermo-mechanical properties of composites making their use in high temperatures and humid environment unfavorable [39, 40].

Although, the results of the present study highlighted the influence of framework and veneer thickness and ratio between them when using different veneering materials for PEEK, yet long-term investigations and further in vivo studies of different veneering materials on PEEK frameworks of anatomical long span fixed dental prosthesis are still recommended. Additionally, the lack of cyclic loading testing is regarded as one of the limitations of the current study. It would have provided better reflection of mechanical performance over an extended period of service.

## Conclusions

Within the limitations of this in vitro study, it could be concluded that manual veneering of PEEK frameworks with conventional composite and using a thicker framework could be more successful than digitally veneered PEEK frameworks with either CAD milled composite or lithium disilicate. However, further interrogation is needed regarding the color stability and the ability of such composite veneered restorations to mimically reproduce the natural teeth esthetics.

## References

[1] Conrad J, Seong W, Pesun I (2007). Current ceramic materials and systems with clinical recommendations: a Systematic Review. J Prosthet Dent..

[2] Stawarczyk B, Beuer F, Wimmer T, Jahn D, Sener B, Roos M, Schmidlin PR (2013). J Biomed Mater Res B Appl Biomater..

[3] Tartuk B, Anna E, Basaran E (2019). Comparison of the load-bearing capacities of monolithic PEEK, zirconia and hybrid ceramic molar crowns. Meanders Med Dent J..

[4] Tekin S, Cangül S, Adıgüzel Ö, Deg˘ er Y. (2018). Areas for use of PEEK material in dentistry. Int Dent Res..

[5] Dal PAMO, Tribst J, Borges A, Souza R, Bottino M (2018). CAD-FEA modelling and analysis of different full crown monolithic restorations. Dent Mater..

[6] Verma A (2018). Novel innovations in dental implant biomaterials science: zirconia and PEEK polymers. Int J Appl Dent Sci.

[7] Jin HY, Teng MH, Wang ZJ, Li X, Liang JY, Wang WX (2019). Comparative evaluation of BioHPP and titanium as a framework veneered with composite resin for implant-supported fixed dental prostheses. Prosthet Dent..

[8] Porojan L, Toma FR, Vasiliu RD, Topală FI, Porojan SD, Matichescu A (2021). Optical properties and color stability of dental PEEK related to artificial ageing and staining. Polymers (Basel)..

[9] Fahmy A, Zohdy M, Abd El Fattah G (2020). Color reproduction of PEEK material veneered with IPS e.max and Visio.lign and composite blocks with different thicknesses. Al-Azhar Journal of Dental. Science..

[10] Sinmazisik G, Demirbas B, Tarcin B (2014). Influence of dentin and core porcelain thickness on the color of fully sintered zirconia ceramic restorations. J Prosthet Dent..

[11] Sloan R, Hollis W, Selecman A, Jain V, Versluis A (2021). Bond strength of lithium disilicate to polyetheretherketone. J Prosthet Dent..

[12] Beleidy M, Ziada A (2020). marginal accuracy and fracture resistance of posterior crowns fabricated from CAD/CAM PEEK cores veneered with HIPC or nanohybrid conventional composite. Egyptian Dental Journal.

[13] Stawarczyk B, Taufall S, Roos M, Schmidlin PR, Lümkemann N (2018). (2018). Bonding of composite resins to PEEK: the influence of adhesive systems and air-abrasion parameters. Clinical oral investigations..

[14] Taufall S, Eichberger M, Schmidlin PR, Stawarczyk B (2016). Fracture load and failure types of different veneered polyetheretherketone fixed dental prostheses. Clin Oral Investig..

[15] Kim JH, Ko KH, Huh YH, Park CJ, Cho LR (2020). Effects of the thickness ratio of zirconia-lithium disilicate bilayered ceramics on the translucency and flexural Strength. J Prosthodont..

[16] Zahran MH, Jokstad A, Tam L, Rizkalla A (2011) Fracture strength of porcelain-zirconia discs: effect of varying thickness ratio. International/American/Canadian Association of Dental Research, 89th general session Abstract number 3212

[17] Xin H, Shepherd D, Dearn K (2013). Strength of poly-ether-ether-ketone: effects of sterilisation and thermal ageing. Polymer Testing..

[18] Özel Bektas Ö, Eren D, Herguner Siso S, Akin G (2012). Effect of thermocycling on the bond strength of composite resin to bur and laser treated composite resin. Lasers. Med. Sci..

[19] BreCAM Consumables. 2018; 1–35

[20] Meshreky M, Halim C, Katamish H (2020). Vertical marginal gap distance of CAD/CAM milled BioHPP PEEK coping veneered by HIPC compared to zirconia coping veneered by CAD-On lithium disilicate “in-vitro study”. Advanced Dental Journal.

[21] Rodríguez V, Tobar C, López-Suárez C, Peláez J, Suárez MJ (2021). Fracture load of metal, zirconia and polyetheretherketone posterior CAD-CAM milled fixed partial denture frameworks. Materials (Basel)..

[22] Shillingburg HT, Hobo S, Whitsett LD (1997). Fundamentals of fixed prosthodontics (ed 3).

[23] Chantranikul N, Salimee P (2015). Biaxial flexural strength of bilayered zirconia using various veneering ceramics. J Adv Prosthodont..

[24] International Organization for Standardization. (2015). Standard No. 6872 for dental polymer and ceramics. https://www.iso.org/standard/59936.html

[25] Shakal MA (2018). Comparative fracture resistance of composite veneered polyether ether ketone crowns with ceramic and composite veneered zirconia crowns. Egyptian Dental Journal..

[26] Darvell W, Gale S (1999). Thermal cycling procedures for laboratory testing of dental restorations. J Dent..

[27] Tay FR, Pashley DH, Yiu CK, Sanares AM, Wei SH (2003). Factors contributing to the incompatibility between simplified-step adhesives and chemically-cured or dual-cured composites. Part I. Single-step self-etching adhesive. J Adhes Dent..

[28] Sachdeva S, Kapoor P, Tamrakar AK, Noor R (2015). Nano-composite dental resins: an overview. Annals of Dental Specialty..

[29] El-Damanhoury HM, Haj-Ali RN, Platt JA (2015). Fracture resistance and microleakage of endocrowns utilizing three CAD-CAM blocks. Oper Dent..

[30] Al-Omari WM, Shadid R, Abu-Naba'a L, El Masoud B (2010). Porcelain fracture resistance of screw-retained, cement-retained, and screw-cement-retained implant-supported metal ceramic posterior crowns. J Prosthodont..

[31] White SN, Miklus VG, McLaren EA, Lang LA, Caputo AA (2005). Flexural strength of a layered zirconia and porcelain dental all-ceramic system. J Prosthet Dent..

[32] Bona AD, Anusavice KJ, DeHoff PH (2003). Weibull analysis and flexural strength of hot-pressed core and veneered ceramic structures. Dent Mater..

[33] Kelly JR, Rungruanganunt P, Hunter B, Vailati F (2010). Development of a clinically validated bulk failure test for ceramic crowns. J Prosthet Dent..

[34] Millen CS, Reuben RL, Ibbetson RJ (2012). The effect of coping/veneer thickness on the fracture toughness and residual stress of implant supported, cement retained zirconia and metal-ceramic crowns. Dent Mater..

[35] Graupner N, Kühn N, Müssig J (2021) Influence of sample thickness, curvature and notches on the Charpy impact strength - An approach to standardise the impact strength of curved test specimens and biological structures. Polymer Testing.:93 ISSN 0142-9418

[36] Scherrer SS, Quinn JB, Quinn GD, Kelly JR (2006). Failure analysis of ceramic clinical cases using qualitative fractography. Int J Prosthodont..

[37] Martos J, Osinaga PW, Oliveira E, Castro LA (2003). Hydrolytic degradation of composite resins: effects on the microhardness. Mater Res..

[38] Souza RO, Özcan M, Michida SM, de Melo RM, Pavanelli CA, Bottino MA, Soares LE, Martin AA (2010). Conversion degree of indirect resin composites and effect of thermocycling on their physical properties. J Prosthodont..

[39] Toledano M, Osorio R, Osorio E, Fuentes V, Prati C, Garcia-Godoy F (2003). Sorption and solubility of resin-based restorative dental materials. J Dent..

[40] Ghavami-Lahiji M, Firouzmanesh M, Bagheri H, Jafarzadeh Kashi TS, Razazpour F, Behroozibakhsh M (2018). The effect of thermocycling on the degree of conversion and mechanical properties of a microhybrid dental resin composite. Restor Dent Endod..

